# The Relationship between Native American Ancestry, Body Mass Index and Diabetes Risk among Mexican-Americans

**DOI:** 10.1371/journal.pone.0141260

**Published:** 2015-10-26

**Authors:** Hao Hu, Chad D. Huff, Yuko Yamamura, Xifeng Wu, Sara S. Strom

**Affiliations:** Department of Epidemiology, University of Texas MD Anderson Cancer Center, Houston, Texas, United States of America; Univerity of Puerto Rico at Mayaguez, UNITED STATES

## Abstract

Higher body mass index (BMI) is a well-established risk factor for type 2 diabetes, and rates of obesity and type 2 diabetes are substantially higher among Mexican-Americans relative to non-Hispanic European Americans. Mexican-Americans are genetically diverse, with a highly variable distribution of Native American, European, and African ancestries. Here, we evaluate the role of Native American ancestry on BMI and diabetes risk in a well-defined Mexican-American population. Participants were randomly selected among individuals residing in the Houston area who are enrolled in the Mexican-American Cohort study. Using a custom Illumina GoldenGate Panel, we genotyped DNA from 4,662 cohort participants for 87 Ancestry-Informative Markers. On average, the participants were of 50.2% Native American ancestry, 42.7% European ancestry and 7.1% African ancestry. Using multivariate linear regression, we found BMI and Native American ancestry were inversely correlated; individuals with <20% Native American ancestry were 2.5 times more likely to be severely obese compared to those with >80% Native American ancestry. Furthermore, we demonstrated an interaction between BMI and Native American ancestry in diabetes risk among women; Native American ancestry was a strong risk factor for diabetes only among overweight and obese women (OR = 1.190 for each 10% increase in Native American ancestry). This study offers new insight into the complex relationship between obesity, genetic ancestry, and their respective effects on diabetes risk. Findings from this study may improve the diabetes risk prediction among Mexican-American individuals thereby facilitating targeted prevention strategies.

## Introduction

The Mexican-American population is a genetically diverse ethnic group with varying proportions of Native American, European and African ancestry. Compared to non-Hispanic European Americans, Native Americans and Hispanic Americans have higher Body Mass Index (BMI), increased rates of obesity, and higher incidence of type 2 diabetes [1–4]. The increased rates of obesity and type 2 diabetes among these populations is due in part to shared environmental risk factors such as lower socio-economical status and diet composition [5–7], but shared genetic risk factors may also be a contributing factor [8–10].

The strong correlation between type 2 diabetes and obesity[11] motivates the hypothesis that Native American genetic ancestry is positively associated with both BMI and type 2 diabetes risk. However, recent studies suggest that this relationship between Native American ancestry, BMI, and type 2 diabetes is far more complex. A study conducted in 170 Hispanic, Native American and mixed-ethnicity students in New Mexico suggested that non-European genetic ancestry was positively correlated with BMI (p = 0.008)[2]. Similarly, a second study of 846 Native American participants recruited from 8 Native American reservations reported a significant positive correlation (p = 1.46e-4) [12], In contrast, a study involving 1,506 Mexican Americans from Starr County, Texas found a negative correlation between Native American ancestry and BMI (p = 0.011)[13]. Each of these studies used genetic markers to estimate individual ancestry and each controlled for socio-economic status. In a sample of Latino populations containing 662 type 2 diabetes cases and 269 controls from central Mexico and Colombia, researchers [6] estimated the amount of European genetic ancestry by genotyping 67 ancestry informative markers (AIM) among participants. They found that non-European ancestry was positively correlated with type 2 diabetes risk (odds ratio of 16.7 and 3.8 for a 100% increase in non-European ancestry in Mexicans and Colombians, respectively). However, after controlling for socioeconomic status, the association between ancestry and type 2 diabetes was much weaker in samples from central Mexico (odds ratio of 6.0, p = 0.02) and non-significant in samples from Colombia. Another study[14] conducted on 286 type 2 diabetes patients and 275 controls from Mexico City found a similar trend: the odds ratio for diabetes associated with a 100% increase in Native American admixture was 1.6, however, this result was not statistically significant. Together, these results suggest that Native American ancestry may increase type 2 diabetes risk, but that the influence of Native American ancestry on BMI values depends on the specific population and the environment. Given the complex relationship among genetic ancestry, BMI, and diabetes risk, we hypothesize that Native American ancestry and BMI influence the risk of diabetes in a non-independent manner. Although some evidence exists for an interaction between BMI and self-reported African ancestry in diabetes risk [15], no such interaction has been reported in Mexican-Americans.

Ultimately, any direct relationship between Native American ancestry, diabetes risk, and BMI should be the result of individual risk variants that are more common in populations with Native American ancestry. Genome-Wide Association Studies (GWAS) have identified over 50 loci significantly associated with diabetes and over 30 loci significantly associated with BMI [16]; at least 14 [9, 17–19] and 10 [8, 20] of them have been replicated in Hispanic populations. Studies in Mexican populations have also identified additional risk loci of diabetes. In 2006, one study on a single Mexican-American family [21] found a novel diabetes risk mutation in *ADRB3* using linkage analysis; more recently, a GWAS study conducted on Mexican-Americans in Starr County, Texas[9] found three novel risk genes, *TOMM7*, *HNT* and *PARD3B*. However, it is largely unknown how much population heterogeneity in diabetes prevalence can be explained by these loci. Encouraging progresses have been made in a 2013 GWAS study in Latin Americans involving 3,848 type 2 diabetes cases and 4,366 controls [10]. A risk haplotype in *SLC16A11* was found to be very common among Native Americans and Mexican Americans but rare in Europeans and Africans; this haplotype accounts for 20% of the difference in type 2 diabetes prevalence between Mexican-Americans and Europeans. However, most of the increased risk of type 2 diabetes in Mexican Americans remains unexplained.

To further investigate the relationship between genetic ancestry, BMI, and diabetes risk among Mexican Americans, we genotyped Ancestry-Informative Markers (AIMs) in 4,662 Mexican American participants living in Houston, Texas. We estimate the genetic ancestry of each individual using ADMIXTURE [22] and then evaluate the relationship between genetic ancestry, BMI, and diabetes risk in this population.

## Results

### Estimating genetic ancestry

We summarized the demographic characteristics of the participants of this study in Table 1, and the information of the 87 AIMs in S1 Table. We first used the tool ADMIXTURE[22] to generate genetic ancestry estimates for each individual. ADMIXTURE is a model-based clustering algorithm for estimating individual genetic ancestry; it uses the same likelihood model as in its predecessor STRUCTURE[23] but is substantially faster[22]. A commonly used approach for estimating genetic ancestry with model-based methods is to calculate the genotype frequencies in the “source” populations using a reference population panel.[24] However, we did not have access to Native American DNA samples or publically available Native American genotype data on all 87 AIMs. Nonetheless, because the likelihood model of ADMIXTURE assumes that all input markers are in linkage equilibrium,[22] only the allele frequency at each individual marker are needed to estimate genetic ancestry. Therefore, we generated the genotypes of 87 AIMs in 3000 simulated individuals of African, Europeans and Native American ancestries, according to previously reported allele frequencies. We then predicted the ancestry proportions of our participants with a supervised-learning strategy using ADMIXTURE.

**Table 1:** Demographic characteristics of participants.

	Men (n = 1273)		Women (n = 3389)		p-value^*^
	Mean	SD	Mean	SD	
Age	40.1	16.8	39.2	14.5	0.163
BMI	29.6	6.1	30.9	7.3	1.41E-06
Overweight (BMI between 25 and 30)	477(37.5%)	-	1011(29.8%)	-	7.81E-07
Obesity (BMI > = 30.0)	525(41.2%)	-	1661(49.0%)	-	2.43E-06
HBP	203(15.9%)	-	595(17.6%)	-	2.06E-01
Diabetes	146(11.5%)	-	445(13.1%)	-	1.38E-01

* p-values are calculated with Wilcoxon rank sum test for age and BMI, and with Fisher exact test otherwise.

**Fig 1:**
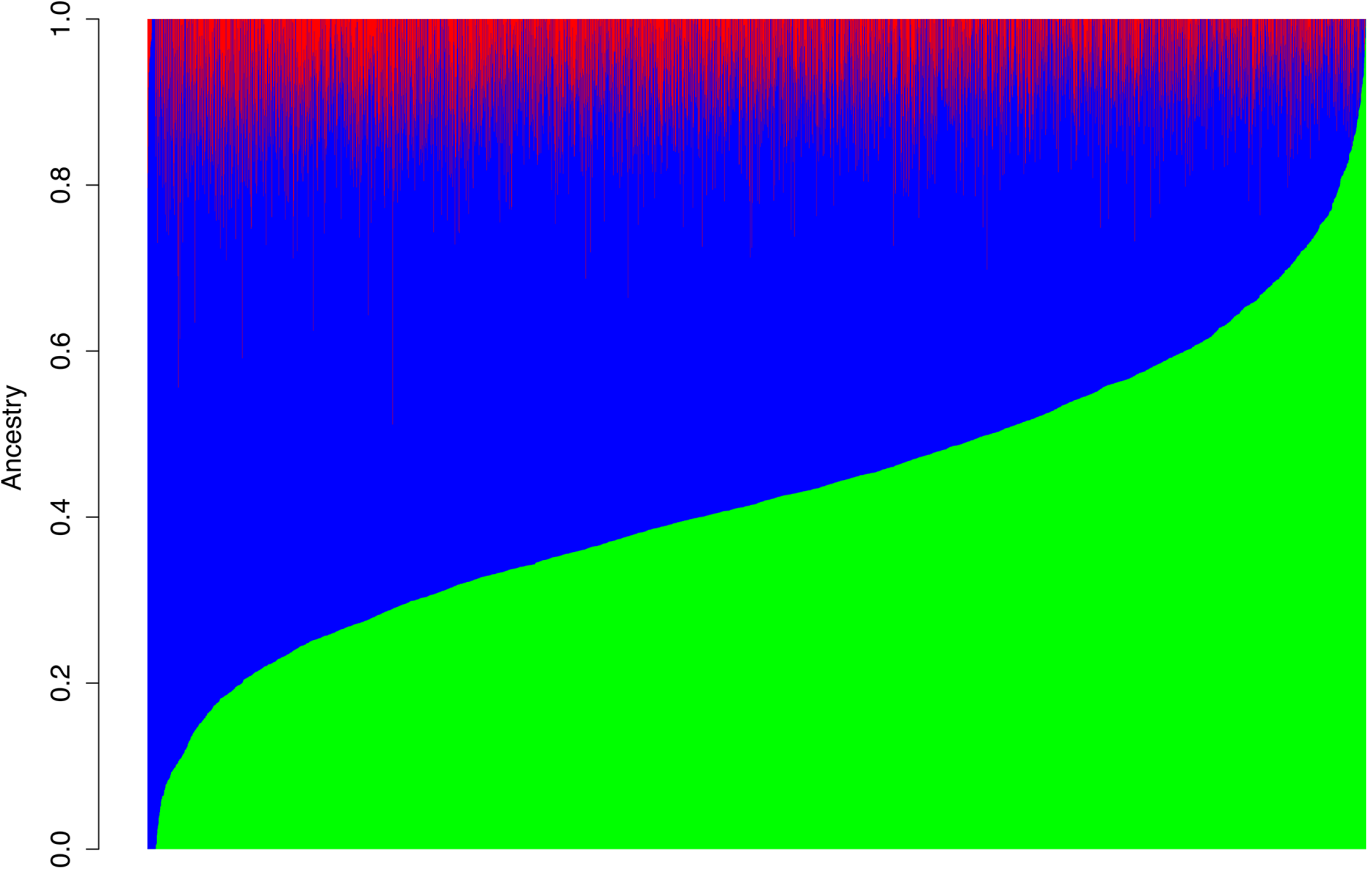
Genetic ancestries of 4,659 participants in the Mexican-American Cohort. Each column represents one individual. The lengths of lines with different colors in each column represent the proportion of genetic ancestry for this individual. Green: European; Blue: Native American; Red: African.

On average, our participants were of 7.1% African ancestry, 50.2% Native American ancestry and 42.7% European ancestry (Fig 1). The variation in African ancestry between individuals was relative small (SD = 0.062) compared to European and Native American ancestries (SD = 0.168 and 0.164, respectively). The composition of genetic ancestry was very similar between men and women (Table 2). In order to validate the genetic ancestry estimate, we also performed a separate ADMIXTURE run and included European and African individuals from 1000 Genomes Projects in the analysis. Our model correctly predicted the ethnicities of these individuals with high accuracies (S2 Table).

**Table 2:** Genetic ancestry by gender.

	Men		Women		p-value^*^
	Mean	SD	Mean	SD	
African	6.91%	6.21%	7.12%	6.23%	0.28
Europeans	43.20%	16.89%	42.50%	16.79%	0.22
Native American	49.90%	16.62%	50.38%	16.37%	0.36

*p-values are calculated with Wilcoxon rank sum test.

### Correlation between genetic ancestry and BMI

**Fig 2:**
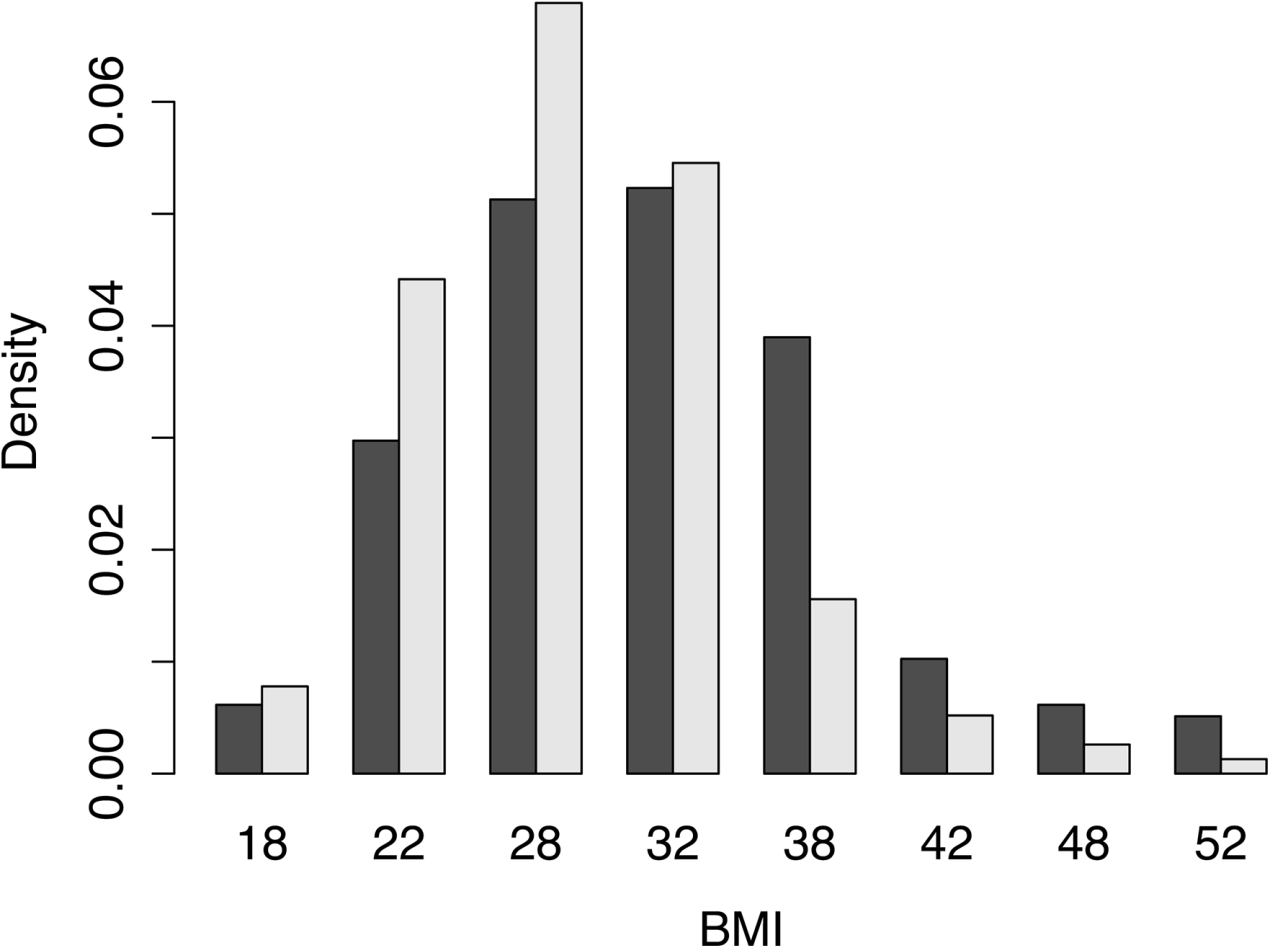
Histogram of BMI values in high-Native American Ancestry group (grey; NA ancestry > 80%) and low-Native American Ancestry group (black; NA ancestry <20%).

We next evaluated the association of genetic ancestry with BMI using multivariate linear regression. Overall, a 10% increase in Native American ancestry reduced BMI by 0.13 m/kg^2^ (Table 3). However, the association was only significant among women, which probably resulted from the smaller sample size in men (n = 1272) versus women (n = 3387). Consistent with this explanation, the confidence interval of the regression coefficient overlapped between men and women, and the interaction term between Native American ancestry and gender was not significant (p>0.1). Additionally, a power analysis indicated that the statistical power to detect a significant effect of Native American ancestry in men is only 25% assuming that the effect size is the same as in women. (Materials and Methods). African ancestry is also significantly correlated with BMI, with a 10% increase in African ancestry increasing BMI by 0.43 m/kg^2^. We next compared the BMI distributions between individuals with less than 20% Native American ancestry (n = 195) and with greater than 80% Native American ancestry (n = 155) (Fig 2) and found that the latter group had significantly lower BMI than the former (p-value = 3.7×10^−4^ from Wilcoxon test), with mean BMI of 29.2 and 31.9, respectively. Remarkably, 30.3% of the individuals from the group with less than 20% Native American ancestry were severely obese (BMI> = 35) compared to 12.3% in the group with greater than 80% Native American ancestry (p = 5.6×10^−5^ from Fisher exact test).

**Table 3:** Association between genetic ancestry and BMI.

	Native American Ancestry		African Ancestry	
	Regression coefficient*	p-value	Regression coefficient*	p-value
Male	-0.29(-2.32,1.74)	0.78	1.62(-3.69,6.92)	0.55
Female	-1.6(-3.15,-0.05)	0.042	5.44(1.49,9.4)	0.0071
Combined	-1.34(-2.60,-0.07)	0.038	4.31(1.05,7.57)	0.01

*Shown in the parentheses are the 95% confidence interval of the regression coefficient

### Association between genetic ancestry, BMI and diabetes status

We evaluated self-reported diabetes status to investigate the influence of Native American ancestry on diabetes risk in Mexican-Americans. When BMI was not included as a covariate, we found that Native American ancestry was a significant risk factor for diabetes, with an OR of 1.067 for a 10% increase in Native American ancestry (p = 0.037; Table 4), which is consistent with previous estimate within American Indian and Alaska Native adults [25]. Interestingly, after controlling for BMI, the association between Native American ancestry and diabetes became stronger (OR = 1.077 for a 10% increase in Native American ancestry; p = 0.019). Combining this with the observation that BMI is significantly lower in Mexican-Americans of high Native American ancestry, we concluded that Native American ancestry increases the diabetes risk despite its inverse association with BMI. When we stratified these data by gender, we found that the association between Native American ancestry and diabetes was only significant among women (Table 4), which once again was likely due to the smaller sample size of men. The 95% confidence interval of the OR for a 10% increase in Native American ancestry was 0.869 to 1.118 in men, and thus the results are consistent with the effect size we observed in women. We also observed no evidence of a Native American ancestry x gender interaction (p = 0.08). In addition, a power analysis suggested that the statistical power to detect a significant effect of Native American ancestry in men is only 38% assuming that the effect size is the same as in women. (Materials and Methods)

**Table 4:** Regression analysis of diabetes status with Native American genetic ancestry.

Not controlling for BMI			Controlling for BMI		
	OR associated with 10% increase in NA ancestry*	p-value		OR associated with 10% increase in NA ancestry*	p-value
Male	0.987(0.871,1.120)	0.84	Male	0.986(0.869,1.118)	0.82
Female	1.097(1.023,1.177)	0.0098	Female	1.108(1.032,1.19)	0.0045
Combined	1.067(1.004,1.135)	0.037	Combined	1.077(1.012,1.145)	0.019

* Shown in the parentheses are the 95% confidence intervals

We next investigated whether the magnitude of risk associated with Native American ancestry differs by BMI level. To address this issue, we stratified the data into four BMI groups based on the clinical guidelines of the National Heart, Lung, and Blood Institute,[26] as shown in Table 5. We analyzed the association between Native American ancestry and diabetes for each group using multivariate logistic regression, after controlling for daily exercise, age, high blood pressure, and acculturation score. Due to the limited sample size and power in men(Table 4), we restricted our analyses to only women. Surprisingly, we found that Native American ancestry was only significantly associated with diabetes risk among overweight and obese women (BMI between 25 and 35), with an OR of 1.190 for a 10% increase in Native American ancestry (p = 0.0004; Table 5). There was no association between Native American ancestry and diabetes risk among severely obese (BMI > = 35) and normal/under- weight (BMI<25) women (p = 0.59 and 0.66; OR = 1.033 and 0.951, respectively). We also performed a likelihood ratio test for interaction between the Native American genetic ancestry and BMI, which was significant at p = 0.049 (See Materials and Methods). These results suggest that Native American ancestry has differential effects on diabetes risk among different BMI groups.

**Table 5:** Regressing Native American genetic ancestry with diabetes risk by BMI in women.

	Sample size	Odds ratio relative to BMI <25 group*	OR associated with 10% increase in NA ancestry*	p-value
BMI < 25	689	1(1,1)	0.951(0.759,1.191)	0.6614
BMI> = 25 and <35	1871	2.248(1.594,3.172)	1.190(1.08,1.311)	0.0004
BMI > = 25 and <30	1011	1.930(1.329,2.801)	1.254(1.081,1.456)	0.0029
BMI> = 30 and <35	860	2.638(1.824,3.814)	1.132(0.993,1.29)	0.0641
BMI > = 35	800	4.233(2.958,6.058)	1.033(0.917,1.163)	0.594
All	3360	-	1.097(1.023,1.177)	0.0098

* Shown in the parentheses are the 95% confidence intervals

## Discussion

In this study, we identified an inverse association between BMI and Native American ancestry in the Mexican American population in Houston, Texas. In contrast, three previous studies [1, 3, 12] reported positive correlations in the Cree population (n = 402), Pima Indians (n = 7,796) and a Native American Community (n = 846). This may suggest an interaction between lifestyle, socioeconomic environment, and genetic ancestry, possibly specific to Mexican Americans. For example, our participants reside in a highly urbanized area of Houston, Texas, in contrast to the indigenous Amerindian community environment in the published data; the differences in the life styles across these populations may contribute to the different directions of association. Interestingly, one study[13] conducted on the Mexican American population in Starr County, Texas (n = 1,506) observed the same inverse association between BMI and Native American ancestry as our study, while two other admixed population studies[6, 24] on native Mexicans (n = 931) and Puerto Ricans living in Boston (n = 1129) did not identify a significant association. Due to our large sample size (4,662) and the homogenous cultural background of our participants, our study was more powerful to detect associations in this admixed population than previous studies. An alternative explanation, however, is that the inverse association between BMI and Native American ancestry only exists in Mexican-American populations. Given the complex relationship between Native American ancestry and diabetes risk, diabetes risk prediction models in populations with Native American ancestry should be carefully calibrated in the target populations.

One possible confounder in the analysis of BMI is diet. High-calorie Western diet characterized by high amounts of red meats, high-fat dietary products and refined grains are reportedly positively correlated with the levels of leptin[27]; therefore, if Native American ancestry is correlated with the diet pattern, then the associations between BMI and Native American ancestry may be confounded. To test this hypothesis, we analyzed a subset of 2,515 respondents who has been asked to rank their diet from 1 to 5, with 1 being “only Mexican foods” and 5 being “only American foods”. We found that the percentage of Native American ancestry were similar across individuals with different dietary patterns (S1 Fig; r^2^ between Native American ancestry and dietary pattern is 0.0086). Also, when we added dietary pattern to the regression model of BMI, the coefficient of dietary pattern was not significant (0.06 (-0.31,0.43); p = 0.74). These evidences suggest that diet is unlikely a confounder in our analyses.

Our results suggest that Native American ancestry may only be an important risk factor for diabetes among overweight and moderately obese individuals. We hypothesize that the Native American ancestry interacts with BMI on diabetes risk in individuals with BMI less than 35, whereas the altered hormonal milieu associated with severe obesity may have an over-arching influence on diabetes relative to the effects associated with Native American ancestry. Interestingly, a previous study [1] had a similar observation in the Cree population, but on the risk of gestational diabetes mellitus. Specifically, normal weight Cree women (≤77kg) have a similar risk of gestational diabetes mellitus as non-Natives women, but overweight Cree (>77kg) women were more than twice as likely to have gestational diabetes compared to obese non-Native whites. Considering the shared etiology between gestational diabetes and type II diabetes[28], it is likely that the same mechanism causes the interaction between BMI and genetic ancestry in both diseases. An alternative explanation is that, because gestational diabetes is also a risk factor of type II diabetes[29], the interaction term between BMI and genetic ancestry in our model is a confounder of the true association between gestational diabetes and diabetes. If this is true, then the odds ratio of the interaction term in gestational diabetes should be much larger than in diabetes. However, this is not the case when we compare [1] to our study (odds ratio of 1.45 for the ethnicity× BMI term in their work and 5.44 for the ethnicity× obesity term in our work), suggesting the latter explanation unlikely.

In our analysis, the Native American ancestry was associated with increased BMI and decreased risk of diabetes only among women but not in men. We attribute this observation to the much larger sample size in women compared to men (2.66 fold), rather than a real difference across the two sexes, for three reasons. First, the confidence intervals of the regression coefficients are overlapping between men and women for both analyses. Second, the power analyses suggested that if we reduced the sample size of the women to the same as men, then we only have 25% and 38% power to detect a significant association of Native American ancestry with BMI and diabetes, respectively. Lastly, a formal Z-test based on the regression coefficients and their standard errors [30] of the Native American ancestry generated a p-value of 0.32 in BMI analysis and 0.11 in diabetes analysis, under the null hypothesis that the effect sizes in men and women are the same. Therefore, we concluded that there is no evidence to support a sex-difference in the risk of BMI and diabetes associated with Native American ancestry.

The main strengths of this study are its large sample size (n = 4,662) and the homogenous cultural background of our participants. The participants are from inner city Houston, therefore reducing variation in environmental factors and increasing statistical power to identify genetic associations. The use of genetic admixture for each individual rather than using self-identification of a complex genetic identity is another strength. We also point out three limitations of this research. First, all cases of diabetes were self-reported and the information on the type of diabetes was not available. Second, information regarding the age of onset was unavailable. Therefore, we used the age at the time of interview as a surrogate for the age of onset during our analyses. Third, because of the high collinearity between Native American and European ancestry, we cannot rule out the possibility that the associations between BMI, diabetes, and Native American ancestry were driven by associations between BMI, diabetes, and European ancestry in the opposite direction. To distinguish between these two possibilities, an independent association study in a population without such high collinearity between Native American and European ancestry is warranted.

In summary, we studied a Mexican American population in Houston, Texas and explored the correlation among Native American ancestry, BMI, and diabetes risk. Interestingly, we found that Native American ancestry was associated with decreased BMI but increased diabetes risk. The increased risk of Native American ancestry for diabetes is most pronounced in overweight and obese individuals but not significant among normal and severely overweight groups, suggesting an interaction effect between BMI and Native American ancestry. Future studies are required to replicate this finding and, if replicated, to reveal the exact mechanism of differential risk. The findings from this study will improve efforts to predict diabetes risk among Mexican-American individuals and facilitate the design of more effective diabetes prevention strategies.

## Materials and Methods

### Study population and sample collection

The study was approved by the institutional review board at M.D. Anderson Cancer Center and all participants have signed a written consent form as per the Declaration of Helsinki. We randomly selected 4,662 individuals who were participants of the Mexican American Cohort, a prospective longitudinal study of residents of Houston, Texas which been previously described.[31] Participants were from a culturally homogeneous but genetically diverse population, making it suited for studying the genetic epidemiology of inherited diseases. We collected demographic, lifestyle, and medical and family history data as well as biological specimens from participants at baseline enrollment. The majority were women (72.7%). In comparison to women, men reported lower BMI on average (29.6 vs. 30.9, p = 1.41x10^-6^ from Wilcoxon rank sum test), and lower rates of obesity (defined as BMI >30; 41.2% vs. 49.0%, p = 2.4x10^-6^ from Fisher exact test). The rates of high blood pressure and diabetes were similar between men and women.

### DNA genotyping and genetic ancestry estimate

We initially selected 96 Ancestry-Informative Markers (AIMs) that have large allele frequency differences among Native Americans, Europeans and Africans, from three different studies on Mexican populations [6, 14, 32]. We included all the AIMs used in the first two studies[6, 14], and a complementary set of 15 AIMs from the third study [32]. We used Illumina GoldenGate Panel to perform the genotyping. Because AIMs within highly admixed populations often deviate from Hardy Weinberg Equilibrium (HWE),[33, 34] we applied a loose HWE filter of p>5x10^-8^. Of the 96 markers, 3 markers had more than 5% missing genotyping rate; 3 were reported as failed calls by the GoldenGate pipeline; 2 were small insertions or deletions (indels); 1 did not pass the HWE filter (S1 Table). These markers were excluded from further analyses. We removed indels because we could not reliably estimate allele frequencies from public sequencing data. In total, 87 markers remained. Three individuals in our data with greater than 5% missing-genotype rate were removed from the study.

Prior to the genetic ancestry calculation, we first estimated the allele frequencies of the 87 AIMs in African (Yoruba population in Ibadan, Nigeria) and European (Utah residents with Northern and Western European ancestry) populations based on the publically available genomes from 1000 Genome Project.[35] For the frequencies of the selected AIMs among Native Americans, we used the reported allele frequencies[6, 14, 32] whenever possible; otherwise, we used the allele frequency estimates in the Totonac population.[36] The 87 AIMs were all separated by at least 600 kb, and the mean and standard deviation of r^2^ value between any two AIMs on the same chromosome was 0.08 and 0.09 in the 1000 Genomes Project.[35] We then simulated genotypes for the 87 AIMs in 1000 individuals from each of the three populations (African, Europeans, and Native Americans) under the assumption that all markers were in linkage equilibrium. Finally, we ran ADMIXTURE [22] on the dataset containing all simulated and genotyped individuals, setting K to 3 and the genetic ancestry of the simulated individuals to 100% of the corresponding population. The ADMIXTURE-predicted ancestry proportions of our genotyped samples were used as the genetic ancestry estimate. We listed the genetic ancestry estimate, diabetes status, BMI and other covariates for each individual in S3 Table.

### Statistical analyses

We used multivariate linear regressions to examine the associations between BMI and genetic ancestry. We included the following covariates that were significantly associated with BMI in our study: daily exercise, age, acculturation score, educational level, percentage lifetime spent in U.S, alcohol consumption, smoking history and African ancestry. We also included educational level in the model because of the documented correlations between educational level and BMI [37]. The Daily exercise covariate is a binary variable indicating whether the participant performs 150 minutes of moderate-intensity activity or 75 minutes vigorous-intensity activity each week. Level of acculturation was assessed with 4 items (language use, linguistic proficiency, TV usage and radio usage) from the Bidimensional Acculturation Scale for Hispanics, a validated instrument that has very good internal consistency (Cronbach's alpha = 0.87) and was designed for use with Mexican Americans[38]. The Educational level covariate is a binary variable indicating whether the participant had at least high-school level education or technical training. The smoking history covariate is a factor that can be "never smoker", "past smoker" or "current smoker". The alcohol consumption covariate was created in a similar fashion. The correlation between African and Native American ancestries was very low (r^2^ = 0.016), which justifies the inclusion of both ancestry estimates in the same model. We did not include European ancestry as a covariate due to its collinearity with Native American ancestry (r^2^ = 0.87).

To analyze the association between diabetes and genetic ancestry, we used multivariate logistic regressions, controlling for the following covariates that were significantly associated with diabetes in our study: daily exercise, age, and high blood pressure status. We also included acculturation score because of the reported association between acculturation score and diabetes. [39] We performed the analysis both with and without the BMI covariate, as indicated in the Results section. For the analyses involving men, we also included alcohol consumption and smoking history, because they were significantly associated with diabetes.

### Power analysis

We have found that the associations between Native American ancestry, BMI, and diabetes were only significant in women but not in men. To investigate whether this was due to the smaller sample size of men (n = 1272) compared to women (n = 3387), we performed the following power analysis. We randomly sampled 1272 individuals among 3387 women with replacement, and on this subset of women constructed the multivariate linear (for BMI analysis) or logistic (for diabetes analysis) regression models in the same way as in the original analyses. We repeated this process 500 times, and recorded what proportion of the re-sampled models reported a significant p-value for Native American ancestry. We used this proportion as the estimate of power for detecting a significant association among men, assuming the effect size is homogenous between men and women.

### Testing the interactions between genetic ancestry and BMI in diabetes risk

In order to evaluate the interaction effect between genetic ancestry and BMI, we first categorized BMI into four groups: 1) normal weight (BMI<25), 2) overweight (BMI between 25 and 30), 3) obese (BMI between 30 and 35), and 4) severely obese (BMI >35). We calculated the relative risk of diabetes for each BMI group using logistic regression, accounting for daily exercise, age, high blood pressure, and acculturation scores. Two neighboring groups (overweight and obese) have similar relative risks and their 95% confidence intervals overlap with each other. Wald tests suggested that the relative risks of these two groups are not significantly different (p = 0.30), while for any other combinations it was always significant. Therefore, we revised our model to merge the overweight and obese participants into one risk group. We then added interaction terms between genetic ancestries and categorical BMI values. This added two degrees of freedom into the previous model; as a result, under the null hypothesis of no interaction, the value of -2 times the log likelihood difference between these two models should be chi-square distributed with 2 degrees of freedom. This allowed us to test whether the interaction term is significant.

## Supporting Information

S1 Fig Boxplot of Native American Ancestry by different dietary pattern groups. Dietary pattern 1 corresponds to “Only Mexican food” and pattern 5 corresponds to “Only American food”.
(EPS)

S1 Table Summary of ancestry informative markers. (XLSX)

S2 Table Proportion of individuals in the 1000 Genome Project corrected predicted by our admixture model under different ancestry fraction cutoffs. (XLSX)

S3 Table Response and predictor variables used for the regression models. (XLSX)
